# Cardiac Involvement in Myotonic Dystrophy Type 1: Mechanisms, Clinical Perspectives, and Emerging Therapeutic Strategies

**DOI:** 10.3390/ijms262210992

**Published:** 2025-11-13

**Authors:** Vamsi Krishna Murthy Ginjupalli, Jean-Baptiste Reisqs, Michael Cupelli, Mohamed Chahine, Mohamed Boutjdir

**Affiliations:** 1 Cardiovascular Research Program, VA New York Harbor Healthcare System, New York, NY 11209, USA; vamsikrishna.ginjupalli@va.gov (V.K.M.G.);; 2 Department of Medicine, Faculty of Medicine, Université Laval, Quebec City, QC G1V 0A6, Canada; 3 CERVO Research Center, Institut Universitaire en Santé Mentale de Québec, Quebec City, QC G1J 2G3, Canada; 4 Departments of Medicine, Cell Biology and Pharmacology, State University of New York Downstate Health Sciences University, New York, NY 11203, USA; 5 Department of Medicine, Cardiology Division, New York University Grossman School of Medicine, New York, NY 10016, USA

**Keywords:** myotonic dystrophy type 1, cardiac electrophysiology, arrhythmias, cardiac conduction defects, preclinical models, therapeutic pipeline, cardiac dysfunction

## Abstract

Myotonic Dystrophy Type 1 (DM1) is a complex multisystemic genetic disorder caused by CTG repeat expansions in the *DMPK* gene, leading to RNA toxicity and widespread splicing defects. These splicing abnormalities affect multiple systems, including the respiratory, skeletal, cardiac, nervous, and endocrine systems, resulting in aggressive symptoms that significantly impact quality of life and survival. Cardiac complications are the second leading cause of deaths in DM1, after respiratory insufficiency. Current research is largely focused on understanding cardiac pathology in DM1. This review highlights recent advancements in the clinical and pathological characterization of DM1 cardiac involvement, preclinical models used to study cardiac dysfunction, and emerging therapeutic strategies that target the molecular basis of DM1. Promising approaches include RNA-targeting strategies such as antisense oligonucleotides (ASOs), gene-editing tools like CRISPR-Cas9, and small molecules that modulate RNA splicing. ASOs aim to reduce toxic RNA accumulation, CRISPR-based approaches aim to excise or correct the expanded CTG repeats, and repurposed small-molecule drugs, such as vorinostat, tideglusib, and metformin, could serve as potential therapeutic agents for DM1 patients with cardiac complications. Despite this progress, several challenges remain: the heterogeneity of cardiac manifestations, unpredictable and often silent progression of arrhythmias, limited therapeutic options beyond implantable cardioverter-defibrillator (ICD)/pacemaker implantations, and complex interplay with the multisystemic nature of DM1. More research and well-designed clinical trials are urgently needed to translate these promising strategies into effective treatments for DM1-associated cardiac disease. Here, we discuss the current knowledge in DM1 cardiac pathology and preclinical models as well as the benefits and pitfalls of the available therapeutic approaches.

## 1. Introduction

Myotonic Dystrophy Type 1 (DM1) is a multi-systemic genetic disorder, also known as Steinert disease, that can effect both children and adults [1,2]. DM1 affects cardiac, skeletal, and smooth muscle as well as endocrine, lens, and nervous systems [3,4]. Clinically, DM1 is characterized by progressive muscle wasting, myotonia, muscle weakness, dysfunction of the esophageal and anal sphincters, and dystrophic changes in skeletal muscle. Identifying cardiac abnormalities in DM1 is a significant challenge due to their often silent and progressive nature. Though DM1 has been known for over a century, there is no widely available treatment.

About 1 in 8000 individuals worldwide are affected by DM1, making it one of the most common form of muscular dystrophy [5]. However, its prevalence varies significantly across different geographical regions due to historical genetic transmission patterns and founder effects [6,7]. Higher prevalence rates have been reported in some regions of Finland and the Québec province in Canada, where the prevalence is up to 1 in 500 individuals [7]. This high prevalence is mainly due to the inheritance of shared ancestral mutation, which carries a 50% chance of transmission to offspring. DM1 is a progressive disorder that worsens in subsequent generations [7,8]. This phenomenon, known as genetic anticipation, leads to progressively larger cytosine, thymine, and guanine (CTG) repeat expansions in in *DMPK* gene, resulting in earlier onset and increased severity of symptoms in descendants [8,9].

Cardiac electrical abnormalities occur in 80% of DM1 patients, and cardiac complications are the second highest cause of death in DM1 patients after respiratory-related causes [10]. The primary cardiac manifestations stem from disruptions in the heart’s conduction systems, frequently resulting in arrhythmias such as conduction blocks and atrial fibrillation (AF). Over time, these abnormalities can progress to heart failure [10,11].

Both ventricular and atrial arrhythmias are common in DM1, with atrial fibrillation, conduction, bundle branch block, and first-degree atrioventricular (AV) block among the most frequently observed [12,13,14,15]. Conduction defects in DM1 tend to worsen over time, often progressing to life-threatening arrhythmias or sudden cardiac death if left untreated. Understanding and managing cardiac abnormalities in DM1 remains challenging due to their silent yet progressive nature [16]. DM1 symptoms are often insidious, with most patients remaining asymptomatic until the disorder has advanced significantly, at which point arrhythmias and conduction defects become clinically apparent [16].

Ambulatory monitoring and electrocardiograms (ECGs), such as Holter monitors and implantable loop recorders, are essential for detecting early electrical abnormalities in DM1 patients [17]. Early detection allows for timely interventions, such as pacemakers and implantable cardioverter–defibrillators (ICDs), which reduce the risk of sudden cardiac death and improve life expectancy [18,19]. Given the multi-systemic nature of DM1, comprehensive therapeutic approaches are needed to address the cardiac complications in DM1. Respiratory insufficiency, diabetes related complications, and metabolic disturbances often worsen cardiac function and increase the risk of arrhythmias [20,21,22,23]. This review aims to explore the cardiac pathology of DM1, discuss the features and limitations of preclinical animal models, and examine both current and emerging treatment strategies.

## 2. Cardiac Clinical Phenotype

Cardiac abnormalities in DM1 are common but vary in severity based on the number of CTG repeats [24,25]. While some individuals experience mild cardiac symptoms, others develop severe, life-threatening complications that worsen prognosis and increase the risk of sudden cardiac death [8,23,26]. However, the precise mechanisms underlying DM1-related cardiac conduction dysfunction remain poorly understood. Abnormalities in the cardiac conduction system often lead to left ventricular dysfunction and arrhythmias, primary manifesting as bundle branch block and AV block, and typically precede symptomatic arrhythmias [27]. Conduction system disease affecting the AV node is recognized as a progressive condition in DM1. Cardiac arrhythmias, particularly complete heart block and ventricular tachycardia, are frequently linked to sudden cardiac death, and atrial flutter and AF significantly contribute to the morbidity and mortality of DM1 [15,28,29]. While atrial arrhythmias may be among the earliest presentations of DM1 [30], left ventricular dysfunction, which increases the likelihood of heart failure, complicates clinical management and heightens the risk of sudden cardiac death [31].

ECG abnormalities are potentially early identifiers of DM1, reflecting disruptions in cardiac conduction pathways. Even asymptomatic DM1 individuals often exhibit prolonged QRS and PR intervals [15]. Prolonged PR intervals in DM1 patients range from 185 ms to 205 ms depending on the length of the CTG repeat, indicating delayed conduction between the atria and ventricles. In contrast, prolonged QRS intervals in DM1 patients are reported between 90 ms and 140 ms indicating delayed intraventricular conduction [15,32,33,34]. Cardiac conduction system abnormalities worsen over time in DM1 patients [15]. Early-stage cardiac manifestations, such as first-degree AV block, often progress to more severe conduction defects, including complete AV block, significantly increasing the risk of sudden cardiac death [15]. His-ventricular interval measured during electrophysiological study can provide more sensitive predictor of major brady arrhythmic events along with PR and QRS intervals [35].

### 2.1. Cardiac Involvement in the Congenital Form of DM1

Patients with the congenital form of DM1 harbor > 1000 CTG repeats within the *DMPK* gene. Congenital DM1 often presents with early-onset cardiac complications. Significant conduction defects like complete AV block along with generalized hypotonia are often detected at birth or in neonatal and early childhood stages. However, cardiac manifestations of childhood and classical DM1 often overlap [36,37]. AV block is a major cause of neonatal mortality in DM1 with rates of 30–40% [36,37]. Congenital DM1 has also been linked to premature births with affected neonates presenting cardiac and skeletal symptoms at birth [38]. Newborns of mothers with DM1 are also at increased risk of transient cardiac abnormalities [39]. These cardiac abnormalities may only be detectable for a short period after birth hence the need for comprehensive pre- and post-natal screening to enable early detection and intervention.

### 2.2. Cardiac Involvement in the Classical Form of DM1

Classical DM1 manifests in young adults with 150 to over 1000 CTG repeats. This phenotype is characterized by progressive conduction abnormalities that increase the risk of cardiac complications like atrial arrhythmias, right or left bundle branch block, and first degree AV block [2,4]. Atrial flutter and AF are common arrhythmias seen in this type of DM1, reflecting progressive conduction system defects that increase the risk of sudden cardiac death and thromboembolic events [27]. Beyond cardiac arrhythmia complications, classical DM1 is also associated with hallmark symptoms like myotonia and muscle weakness [4,40].

### 2.3. Cardiac Involvement in Mild or Late-Onset DM1

Individuals with mild or late-onset DM1 with smaller CTG repeat expansions generally have milder cardiac involvement and complications. Late-onset DM1 often presents with subtle or asymptomatic cardiac conduction defects until later in life [4]. However, despite the milder phenotypes, these individuals are still at risk for progressive conduction system abnormalities as they age. Due to the often asymptomatic nature of the disorder, diagnosis and treatment are frequently delayed, leading to worse outcomes by the time of detection [22]. Clinical phenotypes based on CTG repeat size are summarized in Table 1.

### 2.4. Structural Impairments

In DM1, structural cardiac abnormalities are common and contribute to the disease’s clinical manifestations. 14–20% of DM1 patients have left ventricular abnormalities including hypertrophy, dilatation, and systolic dysfunction [31,51]. Left atrial dilatation is seen in a smaller subset of patients [52]. Valvular abnormalities, including mitral valve prolapse, are present in 13.7% of DM1 patients [52]. Regional wall motion abnormalities and local wall thinning have been frequently reported [51,52]. Myocardial fibrosis detectable by late gadolinium enhancement affects 12.5–13% of patients, mainly in the mid-myocardium of the interventricular septum [51]. Right ventricular involvement is less common but occurs with left ventricular abnormalities. Interstitial fibrosis, fatty infiltration, and myocyte hypertrophy are often seen in the cardiac tissues of DM1 patients [46,47]. These structural changes can be present even in the absence of clinical symptoms and are associated with age, sex, and abnormal ECG findings. Up to 16% of patients with normal ECG recordings may still have underlying myocardial alterations, emphasizing the importance of comprehensive cardiac evaluation in DM1 [51].

### 2.5. Cardiac Autonomic Nervous System Imbalance

The heart undergoes extensive neural innervation during development, which is essential for maintaining normal physiological function. Cardiac autonomic nervous system (ANS) imbalance is a major risk factor for fatal arrhythmias. Heart rate variability (HRV) and heart rate turbulence (HRT) are reliable, non-invasive markers to assess ANS function and predict arrhythmic risk [53]. In DM1 patients, HRV studies consistently show autonomic impairment correlated with disease severity. In a large cohort (*n* = 289), both Standard Deviation of NN intervals (SDNN) and Standard Deviation of the Averages of NN intervals (SDANN) decreased with age and CTG repeat length (≈8 ms per decade or per 500 repeats). Frequency analysis revealed reduced total, low frequency (LF), and high frequency (HF) power, with an increased LF/HF ratio, indicating sympathetic dominance [54]. Another study confirmed reduced HRV during deep breathing and lower LF power, reflecting parasympathetic dysfunction [55]. Overall, DM1 patients exhibit global autonomic reduction involving both sympathetic and parasympathetic branches, even at early disease stages. HRV correlates with age and CTG repeat length similar to skeletal and cardiac abnormalities, with stronger ANS involvement in males [56].

## 3. Pathophysiology and Role of Ion Channels in DM1 Cardiac Abnormalities

**Figure 1:**
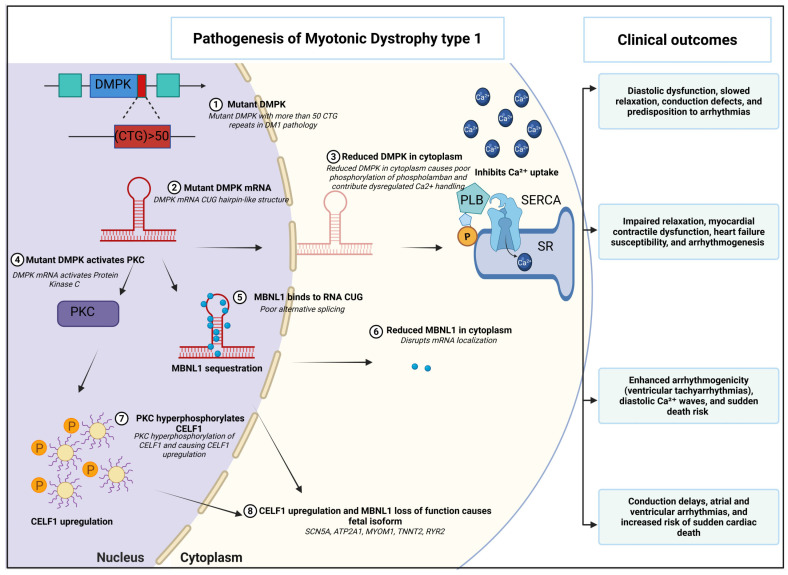
Schematic of molecular derangements driving cardiac pathology in myotonic dystrophy type 1: In DM1, a large CTG repeat expansion (>50 repeats) within the 3′ UTR of the *DMPK* gene (1) gives rise to mutant *DMPK* mRNA forming extended CUG hairpin structures (2). These toxic CUG repeats activate protein kinase C (PKC) (4), which in turn hyper phosphorylates and stabilizes CELF1 (7), leading to upregulation. Simultaneously, CUG hairpin structures sequester MBNL1 into nuclear foci (5), triggering its degradation and depleting both nuclear and cytoplasmic pools of MBNL1 (6). In the cytoplasm, loss of DMPK protein (3) causes poor phosphorylation of phospholamban (PLB) and impairs SERCA-mediated Ca^2+^ uptake. The combined effects of *DMPK* haploinsufficiency, MBNL1 loss of function, and CELF1 gain of function drive mis-splicing and “re-fetalization” of critical cardiac transcripts *SCN5A*, *JUNCTIN/ASPH*, *ATP2A1*, *MYOM1*, *TNNT2*, and *RYR2* (8). These molecular insults culminate in the following pathologies: diastolic dysfunction and slowed relaxation due to defective Ca^2+^ re-sequestration; conduction delays and bundle-branch blocks from mis-spliced ion channels; arrhythmogenesis (atrial and ventricular arrhythmias; diastolic Ca^2+^ waves) driven by Sarcoplasmic Reticulum (SR) Ca^2+^ leak and aberrant channel activity; myocardial contractile dysfunction, heart failure susceptibility; and increased risk of sudden cardiac death.

The expanded CTG repeats in the *DMPK* gene result in the transcription of CUG-expanded mRNAs which form hairpin-like structures that accumulate in the nucleus. These aberrant mRNA structures sequester RNA splicing factors, particularly muscle-blind-like (MBNL) proteins [57], disrupting cellular functions in the heart and muscle tissues and contributing to DM1’s multi-systemic manifestation [58,59,60,61]. MBNL1 and MBNL2 are RNA binding proteins that regulate alternative splicing, RNA stability, and RNA localization, all of which are essential for normal cellular function and development [57]. In DM1, the toxic RNA from the expanded CTG repeats sequesters the MBNL proteins in the nucleus and depletes its function. This leads to widespread splicing defects, altered gene expression, and muscle dysfunction and leading to multi-system disease [62,63,64]. CUGBP Elav-Like Family Member 1 (CELF1), another RNA binding protein critical for normal splicing and muscle function, is also disrupted in DM1. In DM1, CELF1 is functionally active but aberrantly upregulated and hyperactivated through hyperphosphorylation [65,66]. CELF1 regulates the alternative splicing of troponin T (*TNNT2*) pre-mRNA by binding directly to the CUG-expanded splicing enhancers within the transcript. This splicing regulation is impaired in both cardiac and skeletal muscle of DM1 patients [67]. In DM1, CELF1 steady-state levels are increased due to the activation of the protein kinase C (PKC) signaling pathway, which enhances CELF1 hyperphosphorylation and stability [68,69]. However, whether the upregulation of CELF1 is directly caused by enhanced CUG-repeat expression remains unclear. Unlike MBNL1, which associates with hairpin structures, CELF1 does not co-localize with CUG-expanded RNA in nuclear foci; instead, it preferentially binds to single-stranded CUG repeats. This suggests that while CELF1 protein levels are elevated in DM1 patients, MBNL1 is primarily sequestered in nuclear foci. By promoting fetal alternative splicing events due to its overexpression, CELF1 significantly contributes to the restoration of fetal splicing patterns in adult tissue within the context of DM1 pathology [66,70]. Additionally, upregulated CELF1 contributes to the mis-splicing of several genes, including those encoding chloride channels, sodium channels, and the insulin receptor, which are linked to key clinical symptoms of myotonia and type 2 diabetes mellitus (T2DM) [71,72] (Figure 1).

### 3.1. Sodium Channel Dysfunction

The *SCN5A* gene, which encodes the Na_V_1.5 channel essential for the rapid depolarization phase of the cardiac action potential, is significantly affected by splicing abnormalities in DM1 [73]. These abnormalities result in the expression of a fetal isoform of Na_V_1.5 driven by a switch of exon 6B (adult) to exon 6A (fetal). This switch reduces function and changes the kinetics of the channel, resulting in slower and less efficient sodium currents [74]. Exons 6A and 6B are mutually exclusive, coding for segments 3 and 4 of the voltage sensor in domain I of the sodium channel, which are critical for its activity [75,76]. Biophysically, 6A isoform exhibits slower activation and inactivation kinetics, and delayed recovery from inactivation compared to adult exon 6B variant. The net effect is a slower max dV/dt of the action potential and a reduction in conduction, which eventually predisposes to arrhythmias [77,78,79]. Clinically, these molecular abnormalities manifest as prolonged QRS and PR intervals on ECGs, which predispose DM1 patients to conduction blocks and potentially life-threatening arrhythmias [79,80]. The slower sodium currents generated by the abnormal Na_V_1.5 channel create an arrhythmogenic substrate with slow conduction, contributing to both ventricular and atrial arrhythmias [81,82]. Recent in vitro studies using patient-specific induced pluripotent stem cell-derived cardiomyocytes (iPSC-CMs) from DM1 patients have shown that Na_V_1.5 dysfunction reduces current density and slows depolarization and conduction velocity, increasing arrhythmogenic potential [80,83]. These findings support the theoretical mechanisms underlying the clinical symptoms seen in DM1 patients.

### 3.2. Calcium Channel Dysfunction

Ca_V_1.2 channels play a key role in the plateau phase of the cardiac action potential by mediating the L-type calcium current (I_CaL_), which is essential for excitation-contraction coupling in cardiomyocytes [84]. Recent studies have shown that calcium channels can be up or down regulated in DM1, depending on the model used and the patients from which the iPSC-CMs were isolated [80,83]. This complex dysregulation of calcium channel activity contributes to the pathogenesis of cardiac symptoms in DM1, highlighting the molecular complexity of the disease and the potential involvement of calcium channels in cardiac dysfunction [80,83].

Our group recently showed that a DM1 mouse model carrying over 1000 CTG repeats in the human *DMPK* gene (DMSXL) has reduced I_CaL_ and impaired calcium handling in ventricular myocytes compared to wild-type mice [85,86]. These calcium handling abnormalities are well established arrhythmogenic features driven by early calcium release, amplitude alternans, and more prominent calcium sparks. Our group demonstrated that DMSXL cardiomyocytes exhibit diminished maximal intracellular calcium levels, suggesting sarcoplasmic reticulum (SR) calcium depletion likely attributable to leaky ryanodine receptor type 2 (RyR2) channels [86]. The same study also showed calstabin2 depletion in DMSXL mice, supporting the idea that impaired RyR2 closure is the cause of SR calcium loss [86]. Hyperphosphorylation of RyR2 by CaMKII is thought to be the key driver of this arrhythmogenic process, promoting calcium leaks and calcium sparks. These spontaneous release events can trigger premature ventricular contractions in the Purkinje system [86]. Such dysfunctions prolong action potential repolarization and duration, creating an electrophysiological environment that is conducive to delayed afterdepolarizations and early afterdepolarizations [83]. DMPK is a serine/threonine kinase that phosphorylates various substrates within cardiomyocytes. We showed, for the first time, that one of the notable substrates is phospholamban (PLB), a regulatory protein that regulates the activity of the SR calcium ATPase (SERCA). Phosphorylation of PLB by DMPK enhances SERCA’s ability to pump calcium into the SR, thereby facilitating muscle relaxation and contributing to proper cardiac contractility [87,88]. In DM1 pathology, mutant DMPK protein remains in the nucleus and the loss of DMPK activity reduces phosphorylation of PLB, impairing SERCA’s calcium reuptake into the SR (Figure 1). Studies in *DMPK^−/−^* mice show significantly lower basal and β-adrenergic-stimulated PLN-Ser16 phosphorylation, correlating with slower cytosolic calcium decay, diastolic calcium overload, impaired relaxation, and diminished cardiac contractility [87,88,89].

### 3.3. Potassium Channel Dysfunction

Potassium channels are underexplored in the context of DM1 arrhythmogenesis. Emerging evidence suggests they may contribute significantly to the cardiac complications of DM1. For example, iPSC-CM (both atrial- and ventricular-like) models of DM1 showed prolonged action potential duration [80,83]. This is supported by findings in two different DM1 mouse models (LC15 and DMSXL), which showed reduced transient outward potassium current (I_to_) [85,90]. In the LC15 mouse model, which carries 200–400 CTG repeats in the human *DMPK* gene and is a model for late-onset/adult DM1 phenotypes, ventricular myocytes showed prolonged action potential duration at 90% repolarization due to I_to_ reduction, which contributes to the prolonged QRS and QTc intervals in this model [90]. When combined with calcium handling defects, these potassium channel dysfunctions create a synergistic effect and increase the risk of malignant arrhythmias in DM1 patients.

## 4. Indirect Effects on Cardiac Dysfunction

Cardiac complications of DM1 are often compounded by comorbidities like respiratory dysfunction and T2DM, which further worsen DM1-related cardiac abnormalities and overall cardiac outcomes [27].

### 4.1. Type 2 Diabetes Mellitus

T2DM is a comorbidity that worsens DM1-related cardiac dysfunction [91,92,93,94]. A significant proportion of DM1 patients develop T2DM mainly due to endocrine dysfunction and insulin resistance associated with DM1 [92]. This resistance is due to dysregulated alternative splicing of the insulin receptor pre-mRNA, which favors the insulin receptor A (IR-A) isoform. The IR-A isoform has higher affinity for insulin but lower signaling capacity, leading to reduced insulin sensitivity [92,95]. These alternative splicing defects, mediated by sequestration of splicing factors like MBNL1, directly contribute to the abnormalities in insulin receptors [92,95]. The molecular pathogenesis involves impaired downstream insulin signaling characterized by reduced basal phosphorylation of key proteins such as Akt/PKB, p70S6K, GSK3β and ERK1/2 [95]. The concomitance of DM1 and T2DM creates a synergistic pathological environment that independently contributes to cardiovascular dysfunction and accelerates disease progression [96]. In addition to the already discussed multi-systemic effects of DM1 on cardiac function, T2DM plays a major role in accelerating endothelial and atherosclerotic dysfunction, often triggering ischemic heart disease [96]. Chronic hyperglycemia in T2DM further worsens microvascular damage increasing the risk of myocardial infarction due to compromised cardiac perfusion [97,98].

The interplay between DM1 and T2DM accelerates the progression of the disease and worsens left ventricular systolic dysfunction, especially in advanced cases of DM1 where conduction defects and left ventricular systolic dysfunction are common [99]. Mechanisms like reduced myocardial contractility, increased myocardial fibrosis, and increased oxidative stress contribute to rapid progression to heart failure [100]. Pharmacological management is further complicated in patients with both conditions. For example, β-blockers, diuretics, and angiotensin converting enzyme inhibitors (ACE inhibitors) can interact with diabetes medications and mask the adrenergic symptoms of hypoglycemia, complicating diabetes management. Moreover, autonomic neuropathy, which is common in both DM1 and diabetes, increases the risk of sudden cardiac death and complicates cardiac arrhythmia management [94,101]. From a clinical perspective, managing DM1 patients with T2DM requires a collaborative individualized approach to balance the management of cardiac- and T2DM-related complications [101].

### 4.2. Respiratory Dysfunction

Respiratory dysfunction, another major comorbidity in DM1, affects about 50% of patients, especially those with advanced disease. Respiratory muscle weakness is a hallmark of DM1 and often leads to chronic respiratory insufficiency and hypoventilation [102,103,104]. Over time, these issues induce chronic hypoxia, which has far reaching effects on cardiovascular function [103]. Chronic hypoxia triggers several compensatory mechanisms like pulmonary vasoconstriction, sympathetic nervous system activation, and increased cardiac afterload [105,106]. This is particularly concerning for DM1 patients whose left ventricles are already compromised by underlying cardiac conduction defects as the additional strain on the heart accelerates the progression of heart failure.

## 5. Animal Models and In Vitro Studies of DM1

Animal models and in vitro studies have been instrumental in understanding cardiac manifestations in DM1 and provide robust systems to replicate human electrophysiological abnormalities like conduction defects and arrhythmia. Mouse models with expanded CTG repeats in the *DMPK* gene have been particularly useful in this regard. These models mimic various DM1 phenotypes by having different repeat expansion sizes, degree of RNA toxicity, abnormal splicing, and tissue specific expression and allow comprehensive studies of DM1 related cardiac, neurological, and skeletal abnormalities (Table 2).

### 5.1. The DMSXL Mouse Model

DMSXL mice were created by breeding DM300 mice which had a 45 kb human DM1 locus with >300 CTG repeats [112]. Aiming to produce a model of severe, congenital DM1 phenotypes, the high level of intergenerational instability of CTG repeats in DM300 mice was exploited through successive breeding, leading to increased CTG repeats over generations and culminating in the creation of DMSXL mice, which carry over 1000 CTG repeats in the human *DMPK* gene [128,129]. These mice recapitulate several key features of DM1, including the formation of nuclear RNA foci containing expanded CUG repeats in various tissues, mild splicing defects, and physiological abnormalities. DMSXL mice show severe phenotypes such as high mortality, growth retardation, muscle weakness (with abnormal histopathology, reduced muscle strength, and lower motor performance), and cardiac and respiratory problems [112,128,130]. They also show molecular hallmarks of DM1 such as MBNL sequestration [112,128,129]. Notably, DMSXL mice have both peripheral and central nervous system involvement, behavioral deficits, and sleep abnormalities such as excessive daytime sleepiness, which is seen in DM1 patients [131].

From a cardiac perspective, DMSXL mice show several conduction abnormalities similar to DM1 patients, including premature ventricular and atrial contractions and sinus pauses [85,86]. The sodium current in DMSXL mice has a 1.7 fold faster inactivation rate which reduced the maximum upstroke velocity [dV/dt]max of ventricular action potentials compared to wild-type mice [81]. Ventricular myocytes from DMSXL homozygous mice show decreased I_CaL_ density with abnormal gating properties and reduced I_to_ density [85]. Similar ion channel dysfunctions have been seen in other DM1 models including LC15 mice and human derived iPSC-CMs [83,90]. These ion channel dysfunctions translate to significant cardiac problems including sinus bradycardia, conduction defects, and premature ventricular and atrial arrhythmias [85,86]. Flecainide, a sodium channel blocker, has been shown to worsen conduction abnormalities and prolong PR, QRS, and QTc intervals in these mice [81,85,86]. As mentioned above, the RyR2 phosphorylation, increased calcium spark frequency, and prolonged calcium transient times in this mouse model translate to significant arrhythmogenic potential as demonstrated by readily inducible calcium alternans, sustained and non-sustained ventricular tachyarrhythmias, premature ventricular contractions, and sinus block [86]. Overall, the complex electrophysiological and molecular abnormalities in DMSXL mice provide a comprehensive model for DM1 cardiac pathophysiology and a platform for testing therapeutic interventions.

### 5.2. LC15 Mouse Model

The LC15 mouse model was developed as a new transgenic mouse system with cardiac specific expression of expanded CUG-repeat RNA (approx. 200 to 400 repeats) [90]. This model allows the study of moderate RNA toxicity in DM1 phenotypes and provides critical information on cardiac electrophysiological abnormalities in DM1. LC15 mice show significant ECG changes, including prolonged QRS and QTc intervals, but no spontaneous arrhythmias at baseline [90]. However, patch-clamp experiments showed profound ion channel dysfunctions in this model. Ventricular myocytes from LC15 mice show reduced action potential upstroke velocity at physiological pacing and prolonged action potential duration at various stimulation rates (1–9 Hz). Voltage-clamp experiments showed rightward shifts in sodium channel activation and steady-state inactivation and marked reduction in I_to_. When challenged with flecainide, LC15 mice are more susceptible to lethal ventricular arrhythmias, making them a model to study cardiac electrical instability in DM1 [90]. The LC15 model recapitulates key cardiac electrophysiological features of DM1 and is a valuable model to understand the molecular mechanisms of cardiac complications in this disease. However, unlike DMSXL mice, LC15 mice do not show multisystemic DM1 phenotypes. Although LC15 mice have robust CUG-expanded RNA and RNA foci in the heart, overall expression elsewhere is below the pathogenic threshold; myotonia, mis-splicing and muscle wasting are not seen outside of the cardiac compartment. LC15 mice are cardiac specific and are a more focused model for cardiac studies rather than broader systemic investigations.

### 5.3. EpA960 Mouse Model

The EpA960 mouse model is an inducible and heart-specific DM1 model that recapitulates all cardiac features of DM1 [132]. This model was generated using a tamoxifen-inducible Cre-loxP system, a commonly used technique to regulate gene expression in mice that allows for spatial and temporal control of gene expression. The EpA960 model expresses 960 CUG repeats in the *DMPK* 3′ UTR specifically in the heart under α-MHC promoter control [132]. Within 2 weeks of induction, these mice develop severe cardiomyopathy and arrhythmias characterized by dilated cardiomyopathy, left ventricle dilation, systolic and diastolic dysfunction, and progressive conduction defects (prolonged PR intervals and widened QRS complexes). At the molecular level, the model shows rapid disease onset. Nuclear RNA foci are visible within 3–6 h of induction. MBNL1 sequestration and elevated CELF1 and CELF2 protein levels are seen in nuclei with RNA foci. Notably, the timing of CELF1 nuclear accumulation and MBNL colocalization correlates with *TNNT2* splicing changes, suggesting both events contribute to disease pathogenesis. The model also shows mis-regulated alternative splicing of key cardiac genes *TNNT2* and *FXR1H* similar to what is seen in DM1 patients. This was the first model to show CELF1 upregulation in cardiac tissue. The rapid molecular response to CUG repeat expression in this model, including CELF1 upregulation and splicing changes, confirms these are primary mechanisms in DM1 pathogenesis rather than secondary effects of cardiac injury [132]. While this model provides important information on DM1 cardiac pathology, the severe phenotype and rapid disease progression leading to premature death within 2 weeks of induction limits its use for long-term studies of disease progression and therapeutic interventions [133]. Leaky transgene expression, even without induction, introduces variables to studies of tissue-specific or cell-specific disease mechanisms [134]. The very rapid disease onset and progression does not mirror human DM1 and may limit its translational relevance [132].

### 5.4. Tetracycline-Inducible CUG960

The tetracycline-inducible CUG960 mouse model uses a bitransgenic system to express CUG-repeat RNA [127]. The system consists of two transgenic components: the TREDT960I transgene, which contains 960 CUG repeats in human *DMPK* exons 11–15 (controlled by a tetracycline response element and minimal cytomegalovirus (CMV) promoter), and the MHCrtTA transgene which expresses a reverse tetracycline transactivator under the α-MHC promoter. RNA expression is controlled by doxycycline administration. When doxycycline binds to the rtTA protein, the complex activates transcription by binding to the TRE sequence. Withdrawal of doxycycline stops transcription and reverses disease features. This model shows key molecular and physiological features of DM1 cardiac pathology including nuclear RNA foci formation, MBNL protein colocalization, and characteristic splicing defects. Approximately 15% of mice show spontaneous supraventricular arrhythmias during short surface ECG recordings (1.5–2 min). Unlike other models, the CUG960 model shows increased left ventricular posterior wall diameter and a 28% increase in heart weight-to-tibia length ratio, indicating cardiac hypertrophy. The model reproduces cardiac conduction delays and supraventricular arrhythmias seen in DM1 patients. A major advantage of this model is the reversibility of disease features. This Tet-On system allows researchers to have temporal control over disease-causing RNA expression by simple administration or withdrawal of doxycycline-containing chow, making it a valuable tool to study disease progression and recovery mechanisms in DM1. Its inducible nature and longer survival compared to other cardiac DM1 models makes it ideal for studying disease mechanisms and testing therapeutic interventions [127].

### 5.5. DMPK Knockout Mouse Model

The *DMPK* knockout mouse model has provided important insights into DM1 pathogenesis but with some discrepancies between studies [88,107]. Unlike DMSXL, LC15, and other mouse models, this model does not have CTG repeat expansions, which allows the role of *DMPK* depletion alone to be studied. *DMPK* knockout mice develop significant cardiac conduction abnormalities including first-, second-, and third-degree AV block with specific compromise of the AV node and His-Purkinje regions [107,135]. Even heterozygous *DMPK*^+/−^ mice develop first-degree AV block similar to DM1 patients, indicating that cardiac conduction is very sensitive to *DMPK* gene products [135]. Earlier studies reported skeletal myopathy and muscle weakness in this model, but recent studies in different genetic backgrounds found no significant muscle or cardiac dysfunction related to *DMPK* gene deletion [136]. *DMPK* knockout mice do not show significant skeletal muscle abnormalities, further highlighting the distinct contributions of *DMPK* depletion versus RNA toxicity in DM1 pathology [107,136]. These models lack the CTG repeat expansion central to DM1 RNA toxicity and do not develop key disease features such as myotonia, cataracts, or multisystemic involvement; therefore, they have limited use in preclinical testing as they do not recapitulate the full DM1 phenotype.

**Figure 2:**
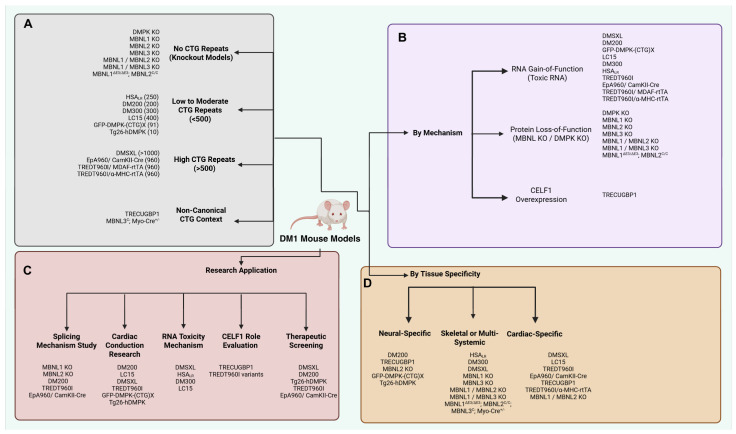
Comprehensive classification of DM1 mouse models by CTG repeat size, pathogenic mechanism, research application, and tissue specificity: (**A**) CTG repeat length. Models are grouped according to the number of CTG repeats in the human *DMPK* transgene (or absence): No CTG repeats (Knockout models): *DMPK* KO; MBNL1, MBNL2, MBNL3 single KOs; compound MBNL1/MBNL2 and MBNL1/MBNL3 KOs; MBNL1^ΔE3/ΔE3^; MBNL2^C/C^; MBNL3^C^; Myo-Cre^+/−^.Low to moderate repeats (<500): HSA_LR_ (∼250); DM200 (∼200); DM300 (∼300); LC15 (∼400); GFP-*DMPK*-(CTG)x (∼91); Tg26-h*DMPK* (∼10). High repeats (>500): DMSXL (>1000); EpA960/CamKII-Cre (960); TREDT960I/MDAF-rtTA (960); TREDT960I/α-MHC-rtTA (960). Non-canonical context: TRE-CUGBP1 (CELF1 overexpression); MBNL3^C^; Myo-Cre^+/−^. (**B**) Pathogenic mechanism. Models are organized by how they recapitulate DM1 molecular pathology: RNA gain-of-function (toxic CUG RNA): DMSXL, DM200, GFP-*DMPK*-(CTG)x, LC15, DM300, HSA_LR_, TREDT960I (all rtTA variants), EpA960/CamKII-Cre. Protein loss-of-function (MBNL or *DMPK* KO): *DMPK* KO; MBNL1, MBNL2, MBNL3 single KOs; MBNL1/MBNL2 KO; MBNL1/MBNL3 KO; MBNL1^ΔE3/ΔE3^; MBNL2^C/C^; MBNL3^C^.CELF1 overexpression: TRECUGBP1. (**C**) Principal research applications. Each model’s most common use in DM1 studies: Splicing mechanism: MBNL KOs; DM200; TREDT960I variants; EpA960/CamKII-Cre. Cardiac conduction research: DM200; LC15; DMSXL; TREDT960I variants; GFP-*DMPK*-(CTG)x; Tg26-h*DMPK*. RNA toxicity mechanism: DMSXL; HSA_LR_; DM300; LC15.CELF1 role evaluation: TRE-CUGBP1; TREDT960I variants. Therapeutic screening: DMSXL; DM200; Tg26-h*DMPK*; TREDT960I variants; EpA960/CamKII-Cre. (**D**) Tissue specificity of perturbation. Models classified by the primary organ system affected: Neural-specific: DM200; TRE-CUGBP1; MBNL2 KO; GFP-*DMPK*-(CTG)x; Tg26-h*DMPK*.Skeletal or multi-systemic: HSA_LR_; DM300; DMSXL; MBNL1 KO; MBNL3 KO; MBNL1/MBNL2 KO; MBNL1/MBNL3 KO; MBNL1^ΔE3/ΔE3^; MBNL2^C/C^; MBNL3^C^; Myo-Cre^+/−^.Cardiac-specific: DMSXL; LC15; TREDT960I variants; EpA960/CamKII-Cre; TREDT960I/α-MHC-rtTA; MBNL1/MBNL2 KO.

Several mouse models specific to cardiac and skeletal muscle involvement in DM1 are listed in Table 2 and Figure 2.

While DMSXL is the most physiologically relevant model that recapitulates multisystemic CTG-driven pathology and authentic mutant human *DMPK* regulation, the choice of platform for cardiac-focused testing depends on the question being asked. For systemic agents (e.g., ASOs or small molecules) that need to penetrate multiple organs, DMSXL is essential for biodistribution, off-target safety, and long-term efficacy. For rapid, cardiac-specific proof-of-concept studies, especially when timing, dose-response, and reversibility need to be evaluated, the tetracycline-inducible CUG960 model is an ideal choice. Its doxycycline-dependent, cardiomyocyte-specific CUG expression reproduces DM1 conduction delays, arrhythmias, and calcium-handling defects and can be turned off to monitor recovery making it the one of the ideal models to test cardiac-targeted DM1 therapies.

### 5.6. In Vitro Studies

In vitro studies using DM1 iPSC-CMs complement the animal models above by providing human-specific insights into molecular mechanisms and splicing defects at the human cellular level. Recent studies using DM1 iPSC-CMs have provided comprehensive insights into cardiac manifestations. Initial studies showed distinct ion channel perturbations in DM1 iPSC-CMs, particularly in cells from a patient with severe cardiac dysfunction and 1300 CTG repeats (DM1-1300), including altered *SCN5A* isoforms, disrupted sodium current activation, and increased I_CaL_ density [80]. Subsequent studies revealed that DM1 iPSC-CMs have intranuclear foci, abnormal transcript splicing, and irregular nuclear morphology due to unbalanced lamin A/C ratio [137]. Recent work has built upon these findings by studying additional patient-derived iPSC-CMs (DM1-1290 and DM1-1640) from DM1 patients with significant cardiac conduction abnormalities, exploring both ventricular and atrial aspects of the disease [83]. These studies showed reduced sodium and I_CaL_ densities, prolonged action potential duration, slower conduction velocity, and impaired calcium transient propagation in both ventricular and atrial cardiomyocytes [83]. These models recapitulate key features of DM1 cardiac pathophysiology including arrhythmogenesis and conduction defects and provide valuable insights into molecular mechanisms underlying both ventricular and atrial involvement in DM1.

DM1 patient-specific iPSC-CMs can be used as a platform to test therapeutic strategies to address RNA toxicity and CTG repeat expansions. Studies on ASO-based therapies and CRISPR-Cas9-based interventions are essential to evaluate their potential to correct molecular defects associated with DM1. Therapeutic trials in DM1 iPSC-CM models could provide critical insights into the feasibility and therapeutic efficiency of RNA-directed approaches to mitigate the molecular abnormalities underlying DM1 cardiac burden [138].

Both animal models and in vitro systems are useful tools to gain clinical insights into DM1 cardiac pathology. Together they provide a complementary view of the molecular mechanisms of DM1. This integrative approach will help to develop and refine RNA-targeted therapies to prevent cardiac complications in DM1 (Table 3).

## 6. Therapeutic Pipeline

The development of therapies for DM1, especially for cardiac defects, is moving fast with many current and emerging options. Advances in molecular biology have led to the design of several promising therapies to restore cellular function by correcting splicing defects and reducing RNA toxicity [140]. These therapies include ASOs, CRISPR-Cas9 gene editing, small interfering RNA (siRNA), and small-molecule therapies. Several pharmaceuticals have moved into later stages of clinical trials, showing that these therapies can greatly impact DM1 management [141].

### 6.1. siRNA and anti-miRNA Molecules

siRNA molecules aim to silence the toxic *DMPK* mRNA through the endogenous RNA interference (RNAi) pathway. After cellular uptake, siRNA duplexes load into the RNA-induced silencing complex (RISC), where the guide strand directs cleavage of complementary CUG-expanded *DMPK* transcripts [142]. AOC 1001 is an antibody-oligonucleotide conjugate composed of a monoclonal antibody that binds to transferrin receptor 1 (TfR1) and is conjugated to a siRNA. This therapeutic approach enables targeted delivery to muscle cells, where the AOC 1001 reduces DMPK transcripts and toxic RNA foci. This allows MBNL proteins to return to their normal splicing functions, restoring healthy splicing variants, and improve muscle function [143,144]. Another siRNA molecule in Phase 1/2a clinical trials is ARO-DM1, developed by Arrowhead Pharmaceuticals (Pasadena, CA). ARO-DM1 is a ligand-conjugated siRNA designed to silence *DMPK* mRNA via an endogenous RNA-interference pathway to reduce the toxic CUG-repeat RNA. ARO-DM1 was evaluated in cynomolgus monkeys for pharmacodynamics and produced robust *DMPK* knockout in skeletal muscle [145]. Although siRNA molecule therapies have shown promise in reducing toxic *DMPK* RNA and correcting RNA-splicing defects, their clinical success will hinge on achieving broad, sustained delivery across cardiac, skeletal, and smooth muscle tissues. Continued refinement of conjugate systems and next-generation chemistries is expected to enhance tissue penetration, durability, and safety.

Anti-miRNA therapies in the DM1 focus on neutralizing the toxic RNA molecules that drive the pathology, rather than altering the mutant *DMPK* transcript. ARTHEx Biotech’s ATX-01 is an anti-miRNA oligonucleotide that inhibits miR-23b, thereby restoring MBNL1 protein production and normalizing splicing activity in DM1 [146]. By reducing RNA foci and rescuing normal splicing activity, these anti-miRNA molecules represent an emerging therapeutic class that complements siRNA approaches to mitigate RNA toxicity in DM1.

### 6.2. Antisense Oligonucleotides

ASOs have emerged as one of the most advanced RNA-targeted therapeutic strategies for DM1. By binding specifically to the mutant *DMPK* transcript, ASOs either promote degradation of the toxic RNA via RNase H activation or sterically block the RNA–MBNL1 interaction, thereby releasing sequestered MBNL proteins [147]. Several DM1 targeting ASOs have been developed using diverse chemical and delivery platforms. The most advanced include ligand-conjugated ASOs from IONIS (IONIS-DMPK-2.5Rx and C16-HA-ASO) (Ionis Pharmaceuticals, Inc., Carlsbad, CA, USA), and antibody-fragment-linked ASO from Dyne therapeutics (Waltham, MA, USA) (DYNE-101).

IONIS-DMPK-2.5Rx was designed to target *DMPK* mRNA to reduce toxic RNA accumulation in cells, especially in cardiac and muscle tissues. As a 16-residue phosphorothioate backbone ASO with 3-10-3 gapmer configuration and cEt modifications, this oligonucleotide targets the 3′-UTR region of *DMPK* mRNA [144,148]. While this ASO approach showed reduction of RNA foci in muscle biopsies at higher doses, ASO concentration in muscle biopsies did not reach the therapeutic threshold. In January 2017, IONIS announced they would not move forward with IONIS-DMPK-2.5Rx due to insufficient target engagement and no significant clinical effect in skeletal muscle tissue. Though this ASO was safe and active in preclinical models, a crucial limitation of naked ASOs is poor muscle penetration, poor endothelial transportation, and poor endosomal escape in myofibers [149]. Then IONIS developed its next generation delivery platform, C16-HA-ASO (Enhanced Ligand-Conjugated ASO), which has a C16-HA ligand to enhance ASO penetration in specific muscle groups. This modification allows the agent to bind and degrade toxic *DMPK* RNA in cardiac and muscle tissues [150]. The improved tissue penetration of C16-HA-ASO resulted in significant reduction of toxic RNA foci and splicing defects, outperforming the original IONIS-DMPK-2.5Rx. C16-HA-ASO has moved beyond early testing and is in preclinical and clinical development to evaluate its efficacy in reducing DM1-related cardiac and skeletal abnormalities [151].

Dyne Therapeutics developed a novel ASO delivery system called the FORCE platform [138]. The FORCE platform uses a human TfR1-targeted Fab fragment conjugated via a cathepsin-cleavable valine citrulline linker to a gapmer ASO against *DMPK* to reduce toxic RNA accumulation in cells. Upon systemic administration, the Fab binds muscle-expressed TfR1 and undergoes receptor-mediated endocytosis; intracellular proteases then cleave the linker, releasing the ASO into the cytoplasm and nucleus. There, RNase H-mediated degradation of expanded CUG-repeat containing *DMPK* transcripts disperses nuclear RNA foci, liberates MBNL splicing factors, and corrects DM1-associated spliceopathy. In both HSA_LR_ and TfR1hu/mu, DMSXLTg mouse models and non-human primates, this approach shows robust *DMPK* knockout, splice correction, and phenotypic improvement with low, infrequent dosing, indicating promise as a systemic DM1 therapy [138]. Since TfR1 is commonly expressed in the most cell types including heart, and the mutant *DMPK* is the central player in DM1 cardiomyopathy, the FORCE platform can deliver ASOs specifically to cardiac and skeletal muscle. By promoting RNase H-mediated clearance of expanded *DMPK* transcripts in the heart, this approach can correct aberrant splicing of calcium-handling genes, such as PLB and SERCA2a, and improve conduction defects and diastolic dysfunction. These ASO strategies highlight the promise and current challenges of targeting *DMPK* for DM1 therapy where improved delivery systems, including peptide, lipid, or antibody conjugates, are critical for achieving effective tissue penetration and durable molecular rescue. Previous studies in *DMPK* knockout mouse models have shown that complete loss of *DMPK* leads to cardiac conduction abnormalities and altered β-adrenergic responses, and its reduced expression suggests that *DMPK* loss impacts conduction and signaling function [135,152].

Klein et al. has developed an ASO, Pip6a-PMO-CAG7, which is specifically designed to target expanded CUG RNA repeats in *DMPK*. Unlike DYNE101 and IONIS-DMPK-2.5Rx, Pip6a-PMO-CAG7 targets only mutant *DMPK* not wild-type allele. The Pip6a peptide enables better penetration of the PMO-CAG7 into muscle and maintains stability in vivo. This ASO reversed splicing defects, normalized the muscle transcriptome, reduced toxic RNA foci, and corrected myotonia [153].

With a similar strategy, VX-670 is a PMO-based ASO therapy that was originally developed by Entrada Therapeutics (ENTR-701; Boston, MA, USA) and is now advanced by Vertex Pharmaceuticals (Boston, MA, USA). VX-670 acts as a steric-block ASO, binding directly to the expanded CUG-repeats in the mutant *DMPK* mRNA [154]. This binding prevents sequestration of MBNL proteins, thereby releasing them to restore normal alternative splicing. These studies support the potential systematic correction of multisystemic DM1 symptoms, including cardiac manifestations.

Together, these studies underscore the therapeutic potential of ASOs in rescuing DM1 cardiac phenotypes and multisystemic manifestations. Ongoing optimization strategies have largely addressed the historical limitation of poor muscle penetration. While DYNE-101 is currently in Phase II clinical trials, VX-670, IONIS, and Pip6a-PMO-CAG7 are under active preclinical investigation.

### 6.3. CRISPR-Cas9 Gene-Editing

CRISPR-Cas9 gene-editing has emerged as a potential solution to the root cause of DM1. As mentioned earlier, the expanded CTG repeats in the mutated *DMPK* produce toxic RNA aggregates that disrupt normal splicing processes, leading to DM1 phenotypes [57]. By targeting the CTG repeats directly, CRISPR-Cas9 offers a permanent solution to RNA toxicity and RNA foci formation. CTG repeat excisions focus on removing or reducing CTG repeat expansions within the *DMPK* gene [155]. By cutting the DNA flanking the expanded repeats, the excision eliminates the formation of toxic RNA, and restores normal function to MBNL proteins [155]. Studies using DM1 patient-derived iPSC-CMs in vitro have shown that successful excision via CRISPR results in improved cellular function [155,156,157].

CRISPR Interference (CRISPRi) on the other hand offers a non-cutting approach to reduce *DMPK* transcription. This approach targets regulatory regions near the CTG repeats to block *DMPK* transcription. This intervention silences *DMPK* expression without cutting DNA and prevents the creation of toxic RNA and splicing abnormalities [158]. Although most previous research has been carried out in skeletal muscle and in vitro cellular studies, the same mechanism of RNA toxicity and spliceopathy leads to conduction defects and arrhythmias in the heart. In principle, CRISPR strategy could normalize the splicing of key cardiac ion channels (e.g., *SCN5A*, *CANA1C*, *KCND3*) and gap junction proteins, which eventually reduce conduction abnormalities and arrhythmogenesis in DM1 patients. However, the major challenge in CRISPR is cardiac-specific delivery, achieving efficient and safe transduction of cardiomyocytes before translating this into a clinical therapy.

### 6.4. Small-Molecule Therapies

Small-molecule therapies offer an alternative approach to DM1 management by stabilizing RNA-protein interactions and modulating RNA splicing to counteract the effects of expanded CTG repeats. Unlike gene-editing technologies and ASO models that target DNA and RNA directly, small molecules work by modulating the cellular pathways that regulate RNA splicing and translation [159].

For example, vorinostat, an FDA-approved histone deacetylase (HDAC) inhibitor, has shown efficacy in modulating epigenetic mechanisms involved in RNA splicing to restore normal splicing patterns in DM1 cells [160,161]. By relaxing chromatin and improving transcriptional accessibility to genes affected by toxic RNA splicing dysregulation, vorinostat can decrease CELF1 activity while increasing MBNL protein expression, thereby correcting spliceopathies in cardiac and muscle cells. Studies in animal models and in vitro DM1 cells have shown that vorinostat can restore splicing for key genes such as *CLCN1* and *SCN5A*, which are involved in cardiac electrophysiology abnormalities in DM1.

Several other FDA-approved drugs are being considered for DM1 treatment plans, including those that target RNA processing and splicing regulations or provide symptomatic relief. Metformin is the most notable repurposed FDA-approved molecule for DM1. Initially approved for T2DM, metformin works through AMP-activated protein kinase (AMPK) pathways that affect cellular metabolism and RNA processing. Activation of AMPK by metformin has been shown to reduce RNA foci and CELF1 [162,163]. The drug mechanism indirectly normalizes splicing in *SCN5A* and rescues genes/channels affected by DM1. Ongoing research and clinical trials have shown that metformin can improve muscle function effectively, and these findings position metformin as a potential therapeutic agent in DM1 management [162]. Another promising small molecule is tideglusib. It is a GSK3β inhibitor and is in development for DM1. In preclinical models, tideglusib showed GSK3β inhibition, improved cellular maturation, and normalized molecular and behavioral features of DM1 [164]. In a more recent study, tideglusib was shown to increase cardiac function by maintaining ejection fraction and fractional shortening in both Ank2-cKO and Dsg2mut/mut mice as these mice contribute to cardiac conduction abnormalities, fibrosis, and sinus node dysfunction. It is also able to rescue cardiac contractile and myocardial fibrosis after phenotype onset. Tideglusib also reduced arrhythmia susceptibility, rescued premature ventricular contractions, and non-sustained ventricular tachycardia [165]. These findings support tideglusib as a promising molecule to rescue cardiac complications in DM1.

Table 4 provides a comprehensive summary of the therapeutic pipeline for DM1, outlining their specific targets, mechanisms, and the latest clinical and preclinical findings. A multitude of compounds, mechanisms, and pathways are leveraged to address both the root cause and phenotype symptoms of DM1. The DM1 cardiomyopathy therapeutic landscape is mature and broad: ASOs are the furthest along as DYNE-101 and IONIS’s ASOs are in advanced clinical trials and show robust target engagement and splice correction in muscle and heart tissue; CRISPR-based approaches, while conceptually powerful, are still in development due to the technical challenges of precisely excising large CTG repeats of mutant *DMPK*, achieving efficient and tissue-specific delivery, and mitigating off-target risks. Importantly, novel and repurposed FDA-approved small-molecule drugs such as HDAC inhibitors and metabolic modulators are ready to be deployed in preclinical and early-phase clinical trials to restore splicing function and alleviate cardiac dysfunction. Together, DM1 therapies form a promising tiered pipeline with ASOs ready for near-term translation, gene-editing tools under long-term development, and currently available small molecules for the interim.

## 7. Conclusions

DM1 is a complex multi-systemic disease that is challenging to manage due to its many symptoms including severe cardiac involvement. Cardiac manifestations, such as conduction defects, arrhythmias, and left ventricular dysfunction, are major causes of morbidity and mortality in affected individuals. These complications, driven by RNA toxicity and ion channel dysfunction, highlight the need for targeted therapies that address the genetic root cause and the multifactorial nature of the disease.

Recent advances in understanding the molecular mechanisms of DM1 cardiac pathology have given us several insights into the role of RNA toxicity, splicing defects, and ion channel dysregulation. At the heart of this pathology is the toxic RNA gain-of-function mechanism driven by the CTG repeat expansion in the *DMPK* gene, which sequesters MBNL proteins and causes widespread splicing defects in key cardiac ion channels including sodium, transient outward potassium, and L-type calcium channels. These disruptions impair normal calcium handling and cause conduction delays leading to prolonged conduction times, arrhythmias, and impaired cardiac contractility. All of these abnormalities increase the risk of sudden cardiac death in DM1 patients, emphasizing the need for targeted therapies.

Recent therapies mark a new era in the management of DM1. Approaches including ASOs, CRISPR-Cas9, and small-molecule therapies are being developed to address specific aspects of DM1’s complex pathology, particularly reducing RNA toxicity and correcting splicing defects. ASOs including IONIS ASOs and DYNE-101 have shown promise in reducing RNA foci in both cardiac and skeletal muscle and improving myotonia. CRISPR-based therapies offer a permanent solution through direct CTG repeat excision with promising results in restoring normal splicing and cardiac function in vitro. However, despite the results, translating CRISPR into the clinical setting is challenging due to specificity, off-target effects, and tissue penetration, particularly in cardiac tissue. More clinical trials and long-term studies are needed to determine the viability of CRISPR as a therapeutic approach for DM1. Beyond the genetic therapies, small-molecule therapies such as vorinostat, tideglusib, and metformin, which modulate RNA splicing or stabilize RNA-protein interactions are another option. While less targeted than ASOs or CRISPR, these are FDA-approved drugs with an established safety record that can be taken orally and affect a wider range of tissues. However, small-molecule therapies also have specificity, side effect, and tissue penetration issues. While these investigations are ongoing, current cardiologists managing DM1 primarily rely on symptomatic and preventative strategies. These strategies include antiarrhythmic drugs for atrial and ventricular arrhythmias, implantation of pacemaker and ICD devices for conduction abnormalities and sudden cardiac death prevention, and regular heart failure treatments including beta blockers, ACE inhibitors, and ARNIs. Careful ECG monitoring and early device implantation remain the cornerstone of cardiac management. While studies to rescue DM1 phenotypes are ongoing, cardiologists are focusing on stabilizing rhythm and function to improve survival and quality of life.

Notably, treating DM1’s cardiac manifestations cannot be done in isolation as comorbidities highly impact the progression of the disease. T2DM is present in a significant proportion of DM1 patients and worsens cardiovascular complications by accelerating left ventricular dysfunction and increasing the risk of myocardial infarction and heart failure. Respiratory dysfunction also contributes to chronic hypoxia, which further stresses the cardiovascular system and increases the risk of arrhythmias and sudden cardiac death. A comprehensive management strategy that includes regular cardiac monitoring, early detection of arrhythmias, aggressive diabetes management, and interventions to mitigate respiratory dysfunction is key to improving long-term outcomes for DM1 patients.

## Figures and Tables

**Table 1:** Classification of DM1 based on CTG repeat size and associated cardiac abnormalities.

Form of DM1	CTG Repeat Range	Onset *	Key Cardiac Features	Skeletal Muscle and Other Systemic Features	Severity	Primary Challenges	References
Mild (Late-Onset) Form	50–150	Middle to late adulthood	Mild conduction abnormalities; occasional first-degree AV block; low incidence of structural abnormalities.	Cataracts, mild myotonia, minimal muscle weakness.	Least severe	Often undiagnosed due to subtle symptoms; may miss early intervention for cardiac monitoring.	[24,27,41,42,43,44,45]
Classical (Adult-Onset) Form	250–500	Late teens to early adulthood	Progressive conduction defects, including PR prolongation and bundle branch block; QTc (corrected QT) prolongation; moderate arrhythmia risk.	Progressive muscle weakness, severe myotonia, cataracts, insulin resistance.	Moderate to severe	Requires ongoing cardiac monitoring due to arrhythmia risk; symptomatic management of muscle issues.	[24,43,44,45,46,47,48,49]
Congenital Form	>1000	Birth or early infancy	Severe conduction delays; AV block, high risk of ventricular arrhythmias; QTc prolongation; structural abnormalities including fibrosis.	Severe hypotonia, respiratory distress, developmental delay, cognitive impairments, dysphagia.	Most severe	Immediate cardiac and respiratory support; early intervention needed for developmental support.	[24,33,45,49,50]
Juvenile Form	500–1000	Childhood to early adolescence	PR and QRS prolongation; moderate QTc prolongation; risk of atrial and ventricular arrhythmias.	Cognitive deficits, myotonia, gastrointestinal issues, mild developmental delay.	Severe	Progressive arrhythmia risk; requires multidisciplinary care to address systemic complications.	[24,45,49]

* Onset is defined as approximate age at which general DM1 symptoms first appear, regardless of the system affected. Abbreviations: Myotonic dystrophy type 1 (DM1), Atrioventricular block (AV block).

**Table 2:** Overview of mouse models developed for myotonic dystrophy type 1 (DM1) research.

Model	Generation Strategy	Phenotype Manifestations	Limitations	Research Application	References
*DMPK* KO	*DMPK* gene deletion	Mild skeletal myopathy and cardiac defects	Does not fully replicate DM1 pathology, particularly RNA gain-of-function effects; potential compensatory mechanisms may mask phenotypes.	Studying the role of *DMPK* in muscle and cardiac function; assessing the impact of *DMPK* loss.	[107,108]
HSA_LR_	Overexpression of human skeletal actin with 250 CTG repeats	Myotonia, muscle weakness, RNA foci formation, splicing defects	Limited to skeletal muscles; does not model for cardiac aspect of DM1	Investigation of RNA toxicity in skeletal muscle.	[109,110]
DM300	Insertion of a 45 kb human genomic fragment containing *DMPK* with 300 CTG repeats	Myotonia, impaired glucose metabolism, muscle atrophy, and RNA foci.	CTG repeat instability in subsequent generations; high mortality; limited splicing alterations	Studying *DMPK* transcription toxicities in tissues.	[111]
DMSXL	Insertion of a 45 kb human genomic fragment containing *DMPK* with >1000 CTG repeats. (Over the generations of DM300)	Motor deficits, RNA foci, MBNL1 sequestration, cerebellar dysfunction, splicing alterations, behavioral abnormalities, cardiac conduction, electrophysiological abnormalities	Decline in transgene expression with age; severe weight loss; high mortality rates	Studying congenital and adult-onset DM1, therapeutic testing	[81,85,86,112]
TREDT960I/MDAF-rtTA	Insertion of a tetracycline-responsive expanded transgene with *DMPK* exon 11–15 transgenes, heart-specific rtTA expression.	RNA foci, MBNL1 sequestration, CELF1 protein upregulation, alternative Splicing alterations, myopathy, and muscle wasting	There is no reproduction of CTG repeat continuity.	Studying and understanding the various mechanisms of CUG-induced muscle wasting.	[113]
EpA960/ CamKII-Cre	Inducible expression of *DMPK* exon 15 with 960 CTG repeats; brain-specific Cre expression	RNA foci, MBNL1 sequestration, CELF1 upregulation, splicing alterations, learning deficits, brain atrophy, neurotransmission dysfunction	Does not reproduce CTG repeat instability; limited to neural tissues	Identifying neural degeneration related to CTG repeat expansions	[114]
DM200	Inducible expression of *DMPK* 3′ UTR with 200 CTG repeats replacing coding sequence with GFP	Cardiac conduction abnormalities, MBNL1 sequestration, RNA foci, and myotonia	Splicing changes in the heart not fully characterized	Investigation of splicing defects and their progression.	[115]
MBNL1 KO	Deletion of MBNL1 exon 3	Splicing abnormalities, myotonia, cataracts	Mild brain alterations; limited spliceopathy compared to DM1	Investigating MBNL1 function, splicing defect studies	[116,117]
MBNL2 KO	Deletion of MBNL2 exon 2	Spatial memory deficits, reduced synaptic plasticity, and splicing alterations	Does not replicate DM1 muscle phenotype	Evaluating MBNL2’s role in splicing regulation and DM1 phenotype	[118]
MBNL3 KO	Deletion of MBNL3 exon 2	Delayed muscle regeneration, neonatal hypotonia	MBNL3 truncation; limited impact on adult muscle function	Assessing functional redundancy among MBNL proteins	[119]
MBNL1/MBNL2 KO	Double knockout of MBNL1 and MBNL2	Myopathy, motor deficits, brain tissue alterations, and skeletal abnormalities.	Reduced lifespan and high mortality before birth.	Evaluating combined loss of MBNL1 and MBNL2 in DM1 muscle phenotype	[120]
MBNL1/MBNL3 KO	Double knockout of MBNL2 and MBNL3 by deleting MBLN1 exon 3 and MBNL exon 2	Impaired chloride conductance, reduced muscle strength, myopathy, and myotonia	Minor brain alterations and limited spliceopathy.	Evaluating combined loss of MBNL1 and MBNL3 in DM1 muscle phenotype	[121]
Mbnl1^ΔE3/ΔE3^; Mbnl2^C//C^; Mbnl3^C^; Myo-Cre^+/−^	Mbnl1 knockout: muscle-specific Cre-mediated MBNL2 and MBNL3 knockout	Spliceopathy, myopathy, muscle wasting, and respiratory difficulties.	Reduced lifespan and high mortality before birth.	Evaluating loss of all the MBNL proteins and their role in DM1 muscular phenotype	[122]
TRECUGBP1	Insertion of a tetracycline-responsive human expressing CELF1 transgene; heart-specific reverse tet trans activator (rtTA)	Splicing alterations and systolic dysfunction.	Limited to cardiac pathology.	Evaluating contribution of CELF 1 expression to DM1 cardiac phenotype.	[123,124]
GFP-*DMPK*-(CTG)X	Overexpression of *DMPK* 3′ UTR with either the wild-type, 11, or expanded, 91, CTG repeats.	RNA foci formation, myotonia, cardiac conduction defects, splicing abnormalities.	Potential for permanent overexpression of human *DMPK*; does not fully replicate multisystemic aspects of DM1.	Understanding the role of *DMPK* expression and RNA toxicity in DM1 pathogenesis; evaluating therapeutic interventions targeting RNA toxicity.	[125]
Tg26-h*DMPK*	Overexpression of human *DMPK* gene in transgenic mice	Myocardial hypertrophy, fibrosis, cardiomyopathy, intracellular calcium overload, reduced blood pressure, and myopathy.	Deficits in chloride channels necessitating use of hyper excitability regulators. Over-expression of hDMPK and increased risks of hypotension. Reduced blood pressure.	Understanding the role of proper expression of hDMPK in ion homeostasis, viability control in muscle cell types, and cytoarchitectural infrastructure.	[126]
TREDT960I/*α*-MHC-rtTA	Insertion of a tetracycline-responsive transgene containing *DMPK* exons 11–15 with 960 interrupted CTG repeats; heart-specific rtTA expression under the α-myosin heavy chain promoter.	RNA foci, MBNL1 sequestration, CELF1 protein upregulation, alternative splicing alterations, arrhythmias.	Does not reproduce CTG repeat continuity; limited to cardiac tissue.	Studying changes in ionic transport in cardio myocytes with CUG toxicities.	[127]
LC15	Insertion of the expanded CTG repeat from the *DMPK* 3′ UTR downstream of a luciferase reporter gene under the control of the CMV-βA promoter.	Prolonged QRS and corrected QT (QTc) intervals, increased susceptibility to ventricular arrhythmias upon flecainide administration, RNA foci formation.	Limited to cardiac defects; does not model multisystemic aspects of DM1.	Evaluating cardiac conduction abnormalities and arrhythmogenic susceptibility in DM1.	[90]

**Table 3:** iPSC-CM Study Models for DM1 and Findings.

DM1 iPSC-CM study	Key Findings	Affected Channels/Genes	Clinical Relevance	References
Spitalieri et al., 2018	Accumulation of RNA foci and MBNL1 sequestration Mis-splicing of *SCN5A* leading to fetal isoform expression Reduced Na^+^ and Ca^2+^ current densities Prolonged action potentials and decreased conduction velocities Impaired calcium transient propagation Observation of arrhythmogenic events	*MBNL1* *MBNL2* *TNNT2* *SCN5A* *CACNA1C* *KCNH2* *KCNQ1* *KCND3*	Recapitulates molecular markers of DM1 Demonstrates altered electrophysiological parameters and biomechanical behavior consistent with unstable cardiac function	[137]
Poulin et al., 2021	Abnormal ion channel functions Slower conduction velocities	*SCN5A* *CACNA1C* *KCNH2*	Highlights the arrhythmogenic potential due to ion channel dysfunction in DM1 cardiomyocytes	[80]
Kim et al., 2019	Presence of MBNL1-positive intranuclear foci Aberrant splicing of target genes Distinct Ca^2+^ transient abnormalities	*MBNL1* *TNNT2* *SCN5A*	Differentiates pathological signatures between DM1 and DM2 Emphasizes the role of MBNL1 sequestration in DM1 cardiac pathology	[139]
Pierre et al., 2023	Accumulation of RNA foci and MBNL1 sequestration Mis-splicing of *SCN5A* leading to fetal isoform expression Reduced Na^+^ and Ca^2+^ current densities Prolonged action potentials and decreased conduction velocities Impaired calcium transient propagation Observation of arrhythmogenic events	*SCN5A* *DMPK* *MBNL1*	Provides insights into molecular and electrophysiological mechanisms underlying DM1 cardiac involvement Highlights the critical role of voltage-gated sodium channels in DM1-related cardiac dysfunctions	[83]

**Table 4:** Summary of Therapeutic Pipeline for DM1.

Therapeutic Class	Drug Candidate	Mechanism	Preclinical/Clinical Model	Current Status	Key Findings	Limitations	References
Small Molecules	Tideglusib	GSK3β inhibitor; reduces RNA foci and normalizes CELF1	HSA_LR_, DMSXL mice; muscle biopsies from patients	Phase III	Improves myotonia, muscle strength, and cognitive symptoms	Limited long-term data and unproven in adults.	[166]
	Metformin	Activates AMPK pathway; modulates glucose metabolism	iPSC-CMs, DM1 patient trials	Phase III	Enhances muscle function and motility	Insufficient multisystem benefit and lack of robust long-term data.	[163,167]
	Pitolisant	Histamine H3 antagonist; targets daytime sleepiness	Clinical trials	Phase II	Reduces excessive daytime sleepiness in DM1 patients	Limited to non-muscular symptoms (EDS, fatigue) and no effect on multisystemic.	[168]
	Mexiletine	Sodium channel blocker; reduces myotonia	Clinical trials	Phase III	Decreases muscle stiffness, improves handgrip strength	GI intolerance, unproven benefit for fatigue and multisystem features.	[169,170]
	Ranolazine	Sodium channel blocker; targets arrhythmias	Clinical trials	Completed Phase I	Limited impact on muscle function, improves heart rhythm	Not recommended to those with existing long QTc and limited long-term safety.	[171,172]
	Flumazenil	GABA receptor modulator; treats cognitive symptoms	Clinical trials	Phase 1	Reduces hypersomnia, improves cognitive function	Short duration of action, primarily targets CNS symptoms, limited availability and access.	[173,174]
	Quercetin	Reduces toxic mRNA levels; exhibits senolytic activity	Cellular and animal models of DM1	Preclinical	Selectively reduces expanded repeat RNA levels and reverses accelerated aging phenotypes in DM1 models	Reversion of benefit and possible cell toxicity at higher doses and Long-term safety unstudied in DM1.	[175]
	Vorinostat	Targets *DMPK* and inhibits mutant *DMPK* levels	HSA_LR_ models	Preclinical	Reduces *DMPK*, rescued MBNL1 sequestration and spliceopathy.	Potential off-target effects, toxicity at higher concentrations, and unknown long-term safety.	[160]
	Erythromycin	Antibiotic; reduces RNA foci accumulation	Cell and mouse models	Phase II	Improves splicing, decreases foci	Modest efficacy and GI side effects for long-term usage.	[176,177]
siRNA molecules	AOC 1001	siRNA targeting *DMPK*; reduces *DMPK* mRNA via TfR1-mediated delivery	Clinical trials	Phase I/II	Reduces *DMPK* mRNA in muscle tissues, corrects splicing	Off-target risks and possible immune response to antibody-oligonucleotide conjugate.	[178]
	ARO-DM1	siRNA is a ligand conjugated via TRiM to target *DMPK*	Clinical trails	Phase I/IIa	Reduces *DMPK* RNA in skeletal muscle.	Off-target risks, unknown safety, and immune response.	[145]
Antisense Oligonucleotides	DYNE-101	ASO conjugated with monoclonal antibody for hTfR1 targeting	Clinical trials	Phase I/II	Reduces *DMPK* RNA in skeletal and cardiac muscle, splicing correction	Unknow long-term safety and moderate side effects.	[179,180]
	IONIS-DMPKRx	ASO; targets *DMPK* mRNA for degradation	DMSXL mouse models	Preclinical	Reduces RNA foci, restores MBNL protein levels	Primarily impacts muscle, not multisystemic and insufficient concentration in muscle.	[150,181,182]
	Pip6a-PMO-CAG7	Peptide-PMO conjugate; targets CUG repeats	HSA_LR_ model	Preclinical	Decreases RNA foci and rescues splicing	Benefit and delivery efficiency in cardiac, CNS tissues unproven.	[153]
	ENTR-701	Peptide-conjugated PMO; blocks CUG repeats	HSA_LR_ model, patient-derived cells	Preclinical/sold to Vertex Therapeutics	Reduces RNA foci, corrects splicing defects	Delivery efficiency unproven, potential immune or off-target.	[183]
	VX-670	Peptide-conjugated PMO; blocks CUG repeats	Clinical trails	Phase I/II	Reduces RNA foci, corrects splicing defects	Efficacy unproven, mechanism and tissue distribution still under study.	[154]
Gene Editing	AAV-CRISPR-SaCas9	CRISPR/Cas9; excises CTG repeats	DMSXL model	Preclinical	Reduces RNA foci, rescues muscle weakness	Very early-stage preclinical trails, delivery efficiency, potential off-target, immune response.	[184,185]
	AAV-PIN-dCas9	dCas9-PIN fusion; degrades toxic RNA	Adult and neonatal HSA_LR_ models	Lead selection	Reduces RNA foci, rescues muscle weakness	Immunogenicity. Delivery efficiency, packaging constraint, off-target effects.	[186]
Anti-miRNA	ATX-01	Inhibit MBNL regulator microRNA-23b (over expresses MBNL)	Clinical trails	Phase I/II	Improves splicing, rescues muscle phenotypes	Immunogenicity and off-target.	[146,187,188]

## Data Availability

The original contributions presented in this study are included in the article. Further inquiries can be directed to the corresponding author.
